# Supplementary material for: Feasibility of an online antigen self-testing strategy for SARS-CoV-2 addressed to health care and education professionals in Catalonia (Spain). The TESTA’T- COVID Project
Source: PLoS One. 2022 Sep 27;17(9):e0275006. doi: 10.1371/journal.pone.0275006 (PMC9514624; doi:10.1371/journal.pone.0275006)
Supplement: Attachment — Submitted filename: Response_to_reviewers_29062022.docx [file pone.0275006.s003.docx]

PONE-D-22-12378

Feasibility of an online antigen self-testing strategy for SARS-CoV-2 addressed to health care and education professionals in Catalonia (Spain). The TESTA’T- COVID Project

COMMENTS TO THE AUTHORS

**ACADEMIC EDITOR:**

Respected authors your manuscript is good. However; your research manuscript needs great attention from your side to reach to required level for publishing specially regarding; the objectives for this study which need to be clear and specific.

**Answer**: Following the recommendations of the Editor and the reviewers, the objectives have been re-written.

The method section has been completed and re-organized in a proper scientific way.

**Answer**:

The results; be sure that your table is inserted within the manuscript and not in the attached other files.

**Answer**: The tables have been inserted within the manuscript.

**Journal Requirements:**

When submitting your revision, we need you to address these additional requirements.

1. Please ensure that your manuscript meets PLOS ONE's style requirements, including those for file naming. The PLOS ONE style templates can be found at
   https://journals.plos.org/plosone/s/file?id=wjVg/PLOSOne_formatting_sample_main_body.pdf and 
   <https://journals.plos.org/plosone/s/file?id=ba62/PLOSOne_formatting_sample_title_authors_affiliations.pdf>

**Answer**:

2. Please provide additional details regarding participant consent. In the ethics statement in the Methods and online submission information, please ensure that you have specified what type you obtained (for instance, written or verbal, and if verbal, how it was documented and witnessed). If your study included minors, state whether you obtained consent from parents or guardians. If the need for consent was waived by the ethics committee, please include this information.

**Answer:** Additional details regarding the informed consent have been included in the new version of the manuscript.

3. Thank you for stating the following in the Competing Interests section: 
"I have read the journal's policy and the authors of this manuscript have the following competing interests: Abbot Laboratories provided the tests for the study for free"

Please confirm that this does not alter your adherence to all PLOS ONE policies on sharing data and materials, by including the following statement: "This does not alter our adherence to  PLOS ONE policies on sharing data and materials.” (as detailed online in our guide for authors http://journals.plos.org/plosone/s/competing-interests).  If there are restrictions on sharing of data and/or materials, please state these. Please note that we cannot proceed with consideration of your article until this information has been declared.

Please include your updated Competing Interests statement in your cover letter; we will change the online submission form on your behalf.

**Answer**: The updated Competing Interests statement has been included in the Competing Interests section and in the cover letter.

4. We note that you have indicated that data from this study are available upon request. PLOS only allows data to be available upon request if there are legal or ethical restrictions on sharing data publicly. For information on unacceptable data access restrictions, please see http://journals.plos.org/plosone/s/data-availability#loc-unacceptable-data-access-restrictions.

In your revised cover letter, please address the following prompts:

a) If there are ethical or legal restrictions on sharing a de-identified data set, please explain them in detail (e.g., data contain potentially identifying or sensitive patient information) and who has imposed them (e.g., an ethics committee). Please also provide contact information for a data access committee, ethics committee, or other institutional body to which data requests may be sent.

b) If there are no restrictions, please upload the minimal anonymized data set necessary to replicate your study findings as either Supporting Information files or to a stable, public repository and provide us with the relevant URLs, DOIs, or accession numbers. Please see http://www.bmj.com/content/340/bmj.c181.long for guidelines on how to de-identify and prepare clinical data for publication. For a list of acceptable repositories, please see http://journals.plos.org/plosone/s/data-availability#loc-recommended-repositories.

We will update your Data Availability statement on your behalf to reflect the information you provide.

**Answer**: In the informed consent approved by the Ethical Committee of the Germans Trias i Pujol Hospital (PI-20-368) it was included the following statement: No sharing data with third party is planned. For this reason, we understand we are not able to share data of the included participants.

5. Please include your tables as part of your main manuscript and remove the individual files. Please note that supplementary tables (should remain/ be uploaded) as separate "supporting information" files.

**Answer**: Done.

**Reviewer's Responses to Questions**

**Comments to the Author**

1. Is the manuscript technically sound, and do the data support the conclusions?

The manuscript must describe a technically sound piece of scientific research with data that supports the conclusions. Experiments must have been conducted rigorously, with appropriate controls, replication, and sample sizes. The conclusions must be drawn appropriately based on the data presented.

Reviewer #1: Partly

Reviewer #2: Partly

**Answer**: We re-wrote the manuscript to solve this issue.

2. Has the statistical analysis been performed appropriately and rigorously?

Reviewer #1: I Don't Know

Reviewer #2: I Don't Know

**Answer**: We further explained the statistical data analysis performed in the new version of the manuscript.

3. Have the authors made all data underlying the findings in their manuscript fully available?

The [PLOS Data policy](https://eur03.safelinks.protection.outlook.com/?url=http%3A%2F%2Fwww.plosone.org%2Fstatic%2Fpolicies.action%23sharing&data=05%7C01%7Ccagusti%40iconcologia.net%7C759d510140f5429ea73308da3f4faee7%7C3b9427dcd30e43bc8c06ff7253676fec%7C1%7C0%7C637891909078619907%7CUnknown%7CTWFpbGZsb3d8eyJWIjoiMC4wLjAwMDAiLCJQIjoiV2luMzIiLCJBTiI6Ik1haWwiLCJXVCI6Mn0%3D%7C3000%7C%7C%7C&sdata=0aLpxPZZiBa1HWIZusdYCAJOqHfFaM1aCF6kGIJ4enc%3D&reserved=0) requires authors to make all data underlying the findings described in their manuscript fully available without restriction, with rare exception (please refer to the Data Availability Statement in the manuscript PDF file). The data should be provided as part of the manuscript or its supporting information, or deposited to a public repository. For example, in addition to summary statistics, the data points behind means, medians and variance measures should be available. If there are restrictions on publicly sharing data—e.g. participant privacy or use of data from a third party—those must be specified.

Reviewer #1: Yes

Reviewer #2: No

**Answer**: In the informed consent approved by the Ethical Committee of the Germans Trias i Pujol Hospital (PI-20-368) it was included the following statement: No sharing data with third party is planned. For this reason, we understand we are not able to share data of the included participants.

4. Is the manuscript presented in an intelligible fashion and written in standard English?

PLOS ONE does not copyedit accepted manuscripts, so the language in submitted articles must be clear, correct, and unambiguous. Any typographical or grammatical errors should be corrected at revision, so please note any specific errors here.

 Reviewer #1: No

Reviewer #2: Yes

**Answer**: We re-wrote the manuscript to solve this issue.

**5. Review Comments to the Author**

Reviewer #1: the respected authors presented a good idea that would add to the available methods of screening. But it is poorly presented, many changes are needed to be suitable for publication.

Abstract: the aim is not properly formulated. Changes will be needed based on the next points.

**Answer**: Following the recommendation of the reviewer, the aim has been reformulated in the abstract and in the introduction section.

Introduction: what is the knowledge gap that you are trying to cross-over.

**Answer**: The introduction section has been extended.

The rationale is missed.

**Answer**: The rationale has been included in the introduction section.

The aim of the study is vague and not fulfilled in the manuscript. You may change it to be:"to assess the feasibility of the TESTATE COVID screening strategy among.......".

**Answer**: Following the recommendation of the reviewer, the aim has been reformulated in the abstract and in the introduction section.

Methods:
Add a figure to illustrate the flow of recruitment process.

**Answer**: Done. See Figure 2.

Move the illustration/definition of the framework components to the methods section.

**Answer**: Done. A figure of the framework components has been included in the methods section and the definition of each component has been added in the text.

What are your study outcomes?

**Answer**: An explanation of what are the study outcomes has been included in the methods section.

The discussion is very confusing, the term "screening" is not properly used. the authors need to clarify what is their target. you may recommend using the kit based on your findings, but in the current status I can't get what is your target.
you need to discuss the main findings, the difference between your 2 groups, and so on ...

**Answer**: We clarified and extended the Discussion section. We agreed with the reviewer that it was not clear enough. We also agreed that the term screening was not properly used and we correct this issue.

The conclusion and recommendation are deficient.

**Answer**: We included more conclusions and recommendations in the Discussion section.

Reviewer #2: The authors have done a good job/
Here are my comments:
Introduction:
- More data from the literature should be mentioned to show the complexity of detecting COVID cases, the use of the antigen tests, and their limitations.

**Answer**: The introduction section has been extended to add more information from the literature to show the complexity of detecting COVID cases, the use of the antigen tests, and their limitations.

- The references used are very limited, more evidence should be added to support the study's claim.

**Answer**: The introduction section has been extended to add more evidence.

Methodology:

- The interpretation of test results by the professional team is not clear.

**Answer**: We added an explanation about the procedure in the Methods section.

- Nothing was mentioned regarding the statistical analysis of data.

**Answer**: An explanation of the statistical analysis of data has been added in the Methodology section of the new version of the manuscript.

- More data regarding inclusion criteria and procedures for collecting the survey data are needed.

**Answer**: An explanation of the inclusion criteria has been added in the Methodology section of the new version of the manuscript

- The used framework for evaluation is not well explained.

**Answer**: An explanation of the framework used for the evaluation has been added in the Methodology section of the new version of the manuscript

Results:

- Adding one or two figures illustrating the data mentioned, especially those from the survey would benefit the presentation of data

**Answer**: An extra table and two figures have been included in the new version of the manuscript.

- The results should be statistically analyzed and presented in the results section

**Answer**: The results section has been modified. We added the p value and the interval of confidence of data presented.

Discussion:

- The discussion section should include more previous studies that are comparable or related to the current study.

**Answer**: Little information is available on pilot intervention that offer self-tests to detect SARS-CoV-2 infection. Some studies have been included in the introduction and the discussion.

- The part of the evaluation should be explained more with relevance to the above-mentioned framework to be able to give a conclusion on the overall feasibility.

**Answer**: The part of the evaluation has been explained more and we included an overall conclusion of the feasibility.

Conclusion:

More conclusions of the study should be added at the end.

**Answer**: More conclusions have been included in the Discussion section.

6. PLOS authors have the option to publish the peer review history of their article ([what does this mean?](https://eur03.safelinks.protection.outlook.com/?url=https%3A%2F%2Fjournals.plos.org%2Fplosone%2Fs%2Feditorial-and-peer-review-process%23loc-peer-review-history&data=05%7C01%7Ccagusti%40iconcologia.net%7C759d510140f5429ea73308da3f4faee7%7C3b9427dcd30e43bc8c06ff7253676fec%7C1%7C0%7C637891909078619907%7CUnknown%7CTWFpbGZsb3d8eyJWIjoiMC4wLjAwMDAiLCJQIjoiV2luMzIiLCJBTiI6Ik1haWwiLCJXVCI6Mn0%3D%7C3000%7C%7C%7C&sdata=s8uU3TOZ0vovdgLEVF6X8gP14pP2aYJC8An4CIoXsUs%3D&reserved=0)). If published, this will include your full peer review and any attached files.

If you choose “no”, your identity will remain anonymous but your review may still be made public.

**Do you want your identity to be public for this peer review?** For information about this choice, including consent withdrawal, please see our [Privacy Policy](https://eur03.safelinks.protection.outlook.com/?url=https%3A%2F%2Fwww.plos.org%2Fprivacy-policy&data=05%7C01%7Ccagusti%40iconcologia.net%7C759d510140f5429ea73308da3f4faee7%7C3b9427dcd30e43bc8c06ff7253676fec%7C1%7C0%7C637891909078619907%7CUnknown%7CTWFpbGZsb3d8eyJWIjoiMC4wLjAwMDAiLCJQIjoiV2luMzIiLCJBTiI6Ik1haWwiLCJXVCI6Mn0%3D%7C3000%7C%7C%7C&sdata=dtAqle2n5zAzM7pYrZ8mWbuVgGy1kJ5FMl4LJkaG2PE%3D&reserved=0).

Reviewer #1: **Yes:** rehab Hosny El-Sokkary

Reviewer #2: No

[NOTE: If reviewer comments were submitted as an attachment file, they will be attached to this email and accessible via the submission site. Please log into your account, locate the manuscript record, and check for the action link "View Attachments". If this link does not appear, there are no attachment files.]
While revising your submission, please upload your figure files to the Preflight Analysis and Conversion Engine (PACE) digital diagnostic tool, [https://pacev2.apexcovantage.com/](https://eur03.safelinks.protection.outlook.com/?url=https%3A%2F%2Fpacev2.apexcovantage.com%2F&data=05%7C01%7Ccagusti%40iconcologia.net%7C759d510140f5429ea73308da3f4faee7%7C3b9427dcd30e43bc8c06ff7253676fec%7C1%7C0%7C637891909078776124%7CUnknown%7CTWFpbGZsb3d8eyJWIjoiMC4wLjAwMDAiLCJQIjoiV2luMzIiLCJBTiI6Ik1haWwiLCJXVCI6Mn0%3D%7C3000%7C%7C%7C&sdata=OPt9vKS7twGhenQVnxSiZib%2FlERyYn8IQoKc9%2BSxiOo%3D&reserved=0). PACE helps ensure that figures meet PLOS requirements. To use PACE, you must first register as a user. Registration is free. Then, login and navigate to the UPLOAD tab, where you will find detailed instructions on how to use the tool. If you encounter any issues or have any questions when using PACE, please email PLOS at [figures@plos.org](mailto:figures@plos.org). Please note that Supporting Information files do not need this step.
